# Preferences of Patients with Non-Communicable Diseases for Primary Healthcare Facilities: A Discrete Choice Experiment in Wuhan, China

**DOI:** 10.3390/ijerph17113987

**Published:** 2020-06-04

**Authors:** Erping Jia, Yuanyuan Gu, Yingying Peng, Xianglin Li, Xiao Shen, Mingzhu Jiang, Juyang Xiong

**Affiliations:** 1 Department of Health Administration, School of Medicine and Health Management, Tongji Medical College, Huazhong University of Science and Technology, Wuhan 430030, Hubei Province, China; m201875310@hust.edu.cn (E.J.); m201875309@hust.edu.cn (Y.P.); m201875311@hust.edu.cn (X.L.); shenxiao0714@hust.edu.cn (X.S.); jiangmingzhu@hust.edu.cn (M.J.); 2 Macquarie University Centre for the Health Economy, Macquarie University, Sydney, NSW 2109, Australia; yuanyuan.gu@mq.edu.au

**Keywords:** discrete choice experiment, non-communicable diseases, primary healthcare facilities, preferences

## Abstract

Objectives: To elicit stated preferences of patients with non-communicable diseases (NCDs) for primary healthcare (PHC) facilities and to explore the willingness-to-pay (WTP) for facility attributes. Methods: A discrete choice experiment (DCE) was conducted through face to face interviews. The DCE survey was constructed by five attributes: type of service, treatment measures, cost, travel time, and care provider. Patients’ preferences and willingness to pay for facility attributes were analyzed using a mixed logit model, and interaction terms were used to assess preference heterogeneity among patients with different sociodemographic characteristics. Results: Patients placed different weights on attributes, depending on whether they perceived their health condition as minor or severe. For conditions perceived as minor, patients valued treatment measures (56.60%), travel time (32.34%) and care provider (8.51%) most. For conditions perceived as severe, they valued treatment measures (52.19%), care provider (38.69%), and type of service (7.30%) most. The WTP related to the change from Traditional Chinese Medicine (TCM) service to Modern Medicine (MM) service was the largest for both severity scenarios. For conditions perceived as minor, patients would be willing to pay 102.84 CNY (15.43 USD) for a reduction in travel time to below 30 min. For conditions perceived as severe, WTP related to the change from general service to specialized service and from junior medical practitioner to senior medical practitioner, were highly valued by respondents, worth 107.3 CNY (16.10 USD) and 565.8 CNY (84.87 USD), respectively. Conclusions: Factors related to the provision of PHC, such as treatment measures, care provider and type of service were highly valued. The findings could contribute to the design of better PHC delivery, improve the participation of patients in PHC, and provide some evidence to promote shared decision-making.

## 1. Introduction

With the acceleration of industrialization, urbanization and aging process, the disease spectrum in China has moved from mainly infectious diseases to the coexistence of infectious diseases and non-communicable diseases (NCDs) [1]. The prevalence of NCDs increased from 15.7% in 2008 to 24.5% in 2013 [2] and attributed to more than 85% of total deaths in 2016 [3]. Burden of NCDs (disability-adjusted life years, DALYs) accounted for 77% of the total disease burden in 2013 [4] and has become a pressing challenge in the public health system in China.

Strengthening primary healthcare (PHC) has been proven to be effective in the prevention and treatment of NCDs [5]. Since health system reforms in 2009, China has made considerable efforts to strengthen the PHC system. The family doctor system, national essential drugs policy, and financial support policy were established or developed for improving medical care at the grassroots level. The number of general practitioners (GP) increased from 109,800 in 2012 to 252,700 in 2017 [6]. The percentage of government subsidies out of total PHC facilities revenue has increased from 12.3% in 2009 to 32.5% in 2017 [7], and the number of PHC facilities increased by 5.77% during the same period [7]. 

Substantial progress has been made through these reforms in the PHC system [8], but most patients tend to bypass PHC and choose higher-level facilities regardless of disease severity [9,10,11]. From 2012 to 2017, outpatient visits to PHC facilities increased by 9.42%, while there was a 33.60% increase for tertiary hospital visits during this period [7,12]. In 2017, the average outpatient cost was 306.1 CNY (45.92USD) at tertiary hospitals, and 117.0 CNY (17.55 USD) at PHC facilities [12]. The overuse of large hospitals has led to rapid increase in medical expenses and heavy economic burden to patients. The underused PHC in China is inconsistent with the World Health Organization’s suggestion that most of common mild illnesses should be treated in PHC facilities [13].

To address these challenges, it is important for policymakers to understand the factors influencing patient preference for PHC facilities. A recent systematic literature review found that empowering the voice of patients in asserting their preferences for healthcare significantly improved prognosis of diseases, facilitated shared decision-making between physicians and patients, as well as promoted uptake and efficiency of healthcare service [14,15]. Other factors impacted patients’ healthcare preference varied with health conditions [16,17,18,19]. Whilst a number of studies examined the general population’s preference for PHC facilities, few have studied patients with NCDs in China. One study examined the factors influencing hypertensive patients’ choices of PHC institutions in Heilongjiang, China [17]. However, it is difficult to know how to prioritize influencing factors due to the lack of information on strength of preference and trade-offs across these factors. It is unclear how much importance should be placed on these factors when patients perceived their health conditions as minor or severe.

The method adopted in our study is discrete choice experiment (DCE), a stated preference survey. It has been proven to be one of the most effective ways to elicit stated preferences [20]. DCEs have been widely used in health economics and policy research and increasingly used to inform the design of healthcare services [20,21,22,23,24]. In this study, we designed a DCE survey to elicit patient preference for PHC facilities and willingness-to-pay (WTP) for attributes and explored the preference heterogeneity across groups with different sociodemographic characteristics in Wuhan, China. 

## 2. Materials and Methods

There are a number of multi-attribute stated preference elicitation methods, but little robust evidence to determine which one is most appropriate [25]. Under the hypothetical task in the case of choosing between alternative treatments, DCE could more accurately reflects the real world decision context than other methods. This method was undertaken in sequential steps following practice guidance [26]: (1) study design development, (2) data collection, and (3) data analysis.

### 2.1. Study Design Development

#### 2.1.1. Selection of Attributes

Identifying relevant preference attributes and levels is key to designing any stated preference study [27]. In the first stage, an initial set of attributes was derived from current health policy documents and literature reviews [16,19,28,29,30,31,32,33]. In the second stage, we recruited five patients with NCDs for in-depth interviews to choose the attributes of the most importance to them. The patients were asked to describe the most important attributes when seeking healthcare and to rank those attributes on the list. In the third stage, the final attributes, levels and wording used in the questionnaire were refined through a focus group with specialists (two directors from two communities, three PHC professionals, and two researchers). The final attributes included three factors related to provision of healthcare, and two factors related to healthcare accessibility and affordability. Cost was included as a value attribute to explore patient trade-off in healthcare attributes. Each of these attributes was then assigned with two or three levels (Table 1). We set disease severity as an impact factor to explore patients’ preferences for healthcare utilization when they perceived their condition as minor or severe [28].

#### 2.1.2. DCE Design

**Figure 1:**
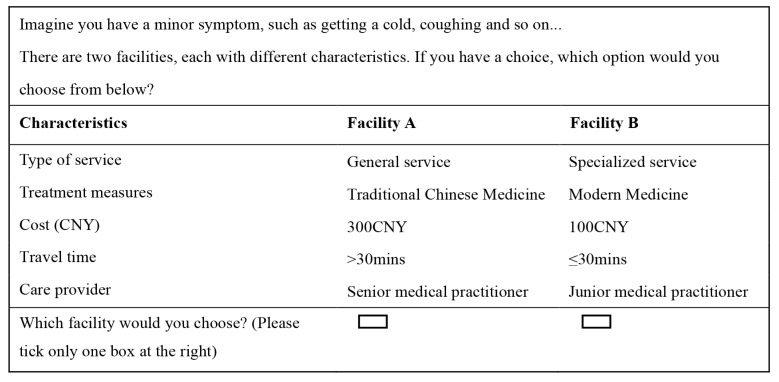
Example of a choice set.

The combination of these attributes and levels (three attributes with two levels, two attributes with three levels) resulted in 72 choice tasks, obviously unfeasible for a survey. We therefore applied orthogonal experimental design (IBM SPSS Statistics 22.0) (IBM, Armonk, NY, USA) to construct 32 choice tasks, and then, divided them into two blocks. Each block formed one version of the questionnaire that contained 16 choice questions and respondents were randomized into these two versions. We included a dominant choice set in the questionnaire to test for rationality before choice questions [14] (i.e., a choice set including one healthcare facility profile characterized by logically preferable levels on all attributes). Given that the uncertainty of respondents’ awareness of levels in the opt-out option and they may choose opt-out option to prevent making difficult choices [34,35], no opt-out option was included (see Figure 1 for a choice set example). A hypothesized disease severity (minor condition or severe condition) was attributed to each choice task. We grouped choice tasks according to the hypothesized severity scenarios (choice tasks under minor conditions were presented first, followed by those under severe conditions). In the beginning of these two groups of choice tasks, there was a brief description of severity scenario. The survey was pretested to 50 patients in Baofeng community in Wuhan in May 2018. After the pilot, the wording and display of the introduction and choice questions were revised.

The final questionnaire consisted of an introductory part (with an overall introduction, explanation of each attribute and example question), the choice set part (choice questions), and the demographic characteristics questions (gender, age, region, marital status, education, employment, family per capita monthly income (CNY)). The questionnaires used in the research are in the online Supplementary File.

### 2.2. Data Collection

Following the sample size calculation methods proposed by Johnson and Orme [36,37], as well as considering the time and budget to conduct survey, we targeted a sample of 180 patients with NCDs aged 45 and over in Wuhan City. Previous studies have indicated that the number of participants is sufficiently large for reliable statistical analysis [38,39]. A pre-defined sample quota on gender (female and male) and region (suburban area and urban area) was used to ensure sample representativeness (Table 2) [40]. According to the stratified random sampling method, we chose two districts, Jiangxia and Qiaokou, which we divided into a low (Jiangxia) and high (Qiaokou) income district based on the gross domestic product by district (see Appendix A) [40]. Then, three PHC facilities were chosen randomly from each district. Finally, we selected thirty patients with NCDs in each PHC facility. 

The local health bureau assigned study coordinators specialized in NCD management from urban and suburban primary health institutions to help us recruit respondents. The study coordinators screened their databases to find eligible participants and contacted them in advance by phone to check their availability to attend the interview. Before data collection, the interviewers were given intensive and uniform training, and were able to explain to the respondents the meaning of choice sets and differences among them. Respondents were invited to PHC facilities to complete the questionnaire anonymously through in-person interviews. They were reminded of making choices under differing disease severity scenarios. The process of administering the questionnaire took about 20 to 25 min on average. The participants were given a gift for their participation. Written informed consent was not obtained from participants, but they were made aware that participation in the research was voluntary and completing a valid questionnaire was an indication of consent. In total, we collected 196 valid questionnaires from May to August in 2018. Ethics approval for the DCE survey was provided by the Committee of Tongji Medical College, Huazhong University of Science and Technology (IORG No: IORG0003571).

### 2.3. Data Analysis

Data analysis was undertaken using Stata 12.0 (Stata Corp LLC, College Station, TX, USA). Respondents characteristics were summarized using descriptive statistics. To determine the relative importance of attributes and to explain preference heterogeneity, we used a mixed logit model based on the random utility theory framework [26]. The utility (U) individual i derived from choosing alternative j may be specified as below:

Uij = Vij (Xij, β) + εij; I = 1, …, n; j =1,2(1)

where Xij is a vector of observed attributes of alternative j, β is a vector of individual specific coefficients reflecting the desirability of the attributes and εij is a random error term. Among the five included DCE attributes, the cost attribute was coded as a continuous variable and all other attributes were effect-coded to represent categorical variables. The coefficient of the reference level for each effect-coded attribute can be calculated as the negative sum of the coefficients of the other levels [41,42]. All coefficients were specified as random except for the one for cost [43]. We set cost as a fixed coefficient, which allowed for an easy derivation and interpretation of the distribution of WTP [43,44].

For this study, we estimated the main effects of the mixed logit model in the first stage. In the second stage, we estimated models with interaction terms to assess potential differences in preferences across groups with different sociodemographic characteristics including gender, region, marital status and so on. The respondent characteristics were mainly binary-coded (see Appendix B). The interaction terms were treated as fixed effect parameters, while main attributes’ coefficients were random except cost [19].

To explore the trade-off among attributes, the monetary value for other attributes was calculated by the ratios between the coefficients of cost with the other non-monetary attributes. The calculation of WTP and confidence interval (CI) was estimated using the delta method [45]. 

## 3. Results

### 3.1. General Characteristics

A total of 196 patients with NCDs aged 45 and over were included in the research. For these participants, age averaged 67 years, with family per capita monthly income of 3000 CNY (450 USD) and 115 (58.7%) patients residing in urban area, and 67.4% female. More than 85% of participants are married, and 51% participants have a middle school qualification. The majority (60.20%) are retired (Table 2).

### 3.2. Preferences under Different Hypothetical Disease Severities

Table 3 showed the DCE results of the two hypothetical severity scenarios. At least one level of the attribute being statistically significant indicates that attributes included in the DCE design would play a critical role in PHC decisions [21]. Almost all of the mean coefficients of attributes were statistically significant and were of the expected sign. For conditions perceived as minor, three of five attributes were significant: treatment measures, travel time, and cost. Patients preferred low price Modern Medicine (MM) service and were concerned about convenient access to PHC facilities (more sensitive to the travel time). From the standard deviation of the regression coefficients, we find that although treatment measures and travel time influence the healthcare choices of patients significantly, the preferences over these attributes vary among respondents. 

For conditions perceived as severe, all attributes have significant impact, except travel time. Patients preferred MM services provided by a senior medical practitioner. The strength and direction of preferences for a senior medical practitioner is next to preferences for MM services. With respect to standard deviation of the regression coefficients, all these non-monetary factors varied except Integration of Traditional Chinese Medicine and Modern Medicine (Integration of TCM and MM). For both hypothetical severity scenarios, the negative signs of cost indicate that patients preferred healthcare that consumes less expenditure. 

### 3.3. Preference Weights and Relative Importance of Attributes and Levels

**Figure 2:**
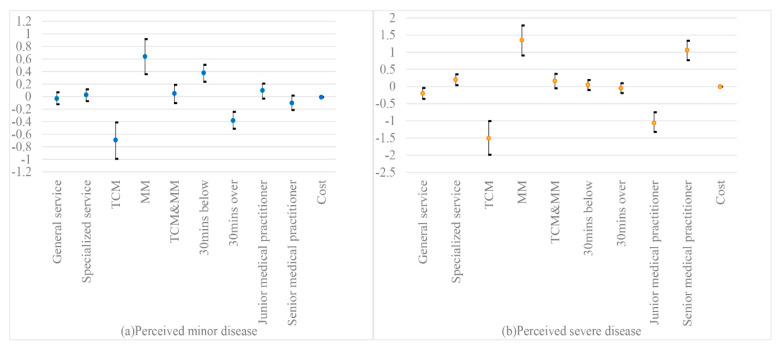
Preference weights with a 95% CI.

The preference weights are illustrated in Figure 2 [46]. The black brackets around each level mean estimate denoted the 95% CI. For conditions perceived as minor, patients’ strongest positive preference was MM service (β = 0.64 (SE = 0.14); *p* < 0.01), followed by travel time from home to PHC facilities below 30 min (β = 0.38 (SE = 0.07); *p* < 0.01). For conditions perceived as severe, patients gave most importance to MM services (β = 1.35 (SE = 0.22); *p* < 0.01), followed by senior medical practitioners (β = 1.06 (SE = 0.14); *p* < 0.01). They also had stronger preferences for specialized service (β = 0.20 (SE = 0.08); *p* < 0.05).

**Figure 3:**
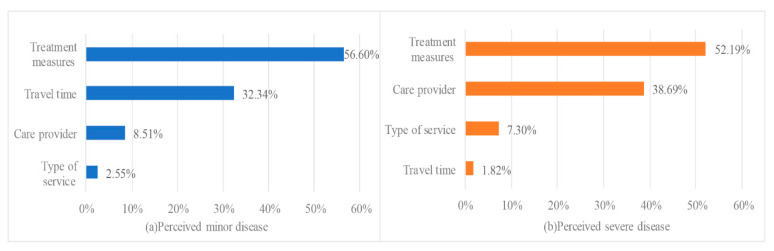
Relative importance of the attributes.

Figure 3 illustrates the relative importance of the attributes. The relative importance for each attribute was determined by ratio of the difference between the lowest and highest level of that attribute, and divided by sum of the difference of all attributes [47,48,49]. For conditions perceived as minor, patients gave the most importance to treatment measures (56.60%), followed by travel time (32.34%), care provider (8.51%) and type of service (2.55%). In contrast, in the severe scenario, patients attached highest importance to treatment measures (52.19%), followed by care provider (38.69%), type of service (7.30%) and travel time (1.82%).

### 3.4. Preference Heterogeneity

Preference heterogeneity analysis in different hypothetical severity scenarios are in Table 4. For conditions perceived as minor, three demographic attributes showed significant influence on healthcare decisions: region, gender and education. The negative coefficient of the interaction between region and cost/travel time indicated that patients living in suburban areas placed less weight on cost and travel time beyond 30 min than their urban residing counterparts. Female respondents experienced less utility for senior medical practitioners compared with their male counterparts. The respondents with a higher education level valued specialized service more than those with a lower education level.

Five out of seven demographic attributes played significant roles in the preferences in the severe disease scenario: marital status, family per capita monthly income, region, gender, and employment. The respondents with higher family per capita monthly income valued cost more than lower family per capita monthly income counterparts. Those respondents who had married placed less weight on cost but larger weight on senior medical practitioners than those single respondents. The respondents residing in suburban areas attached more importance on senior medical practitioners and specialized service. Employed respondents placed higher importance on Integration of TCM&MM than those who were unemployed. Female patients were not inclined to MM services compared with male patients.

### 3.5. WTP Estimates

Table 3 presents the WTP of the attribute levels and their 95% CI. WTP are derived from the mixed logit model without interactions. The WTP results are the amount of cost that respondents would be willing to pay from a worse level to a better level within an attribute. For both severity scenarios, the WTP related to the change from TCM service to MM service was the largest. Respondents would be willing to pay almost 181.75 CNY (27.26 USD) to change from TCM service to MM service under minor condition, while this WTP would increase to 761.73 CNY (114.26 USD) under severe disease scenario. For conditions perceived as minor, they would be willing to pay 102.84 CNY (15.43 USD) for a reduction in travel time to below 30 min. Under a severe condition, WTP related to the change from general service to specialized service and from junior medical practitioner to senior medical practitioner were highly valued by respondents, worth 107.3 CNY (16.10 USD) and 565.8 CNY (84.87 USD), respectively.

## 4. Discussion

To our best knowledge, this is the first study to elicit and quantify PHC facility preferences of patients with NCDs in China. Patients placed different weights on attributes, depending on whether they perceived their conditions as minor or severe. For conditions perceived as minor, patients valued treatment measures (56.60%), travel time (32.34%) and care provider (8.51%) most. For conditions perceived as severe, they valued treatment measures (52.19%), care provider (38.69%), and type of service (7.30%) most. The mixed logit model estimates further suggested the existence of preference heterogeneity among patients with different sociodemographic characteristics. All in all, patients preferred MM services at a lower price and were concerned about convenient access to PHC facilities (more sensitive to the travel time) when they perceived their conditions as minor. All attributes have significant impact when they perceived their conditions as severe except travel time. Patients preferred MM healthcare provided by senior medical practitioners. It is worth noting that although health expense plays a role in healthcare alternatives, it is a relatively minor factor when health status was worse-off. 

These findings are consistent with the literature, which demonstrated that treatment measures were the most important factors that affected healthcare seeking. For both severity scenarios, the WTP related to the change from TCM service to MM service was the largest. Respondents would be willing to pay almost 181.75 CNY (27.26 USD) to change from TCM service to MM service under minor condition, while this WTP would increase to 761.73 CNY (114.26 USD) under severe disease scenario. A survey was conducted among 527 community-dwelling residents in Pudong, Shanghai. Although having a 94.1% awareness rate of TCM, only 48.6% of community-dwelling residents utilized TCM services in actual behavior [50]. Previous studies also confirmed that although TCM for prevention and treatment is acknowledged and used increasingly by residents, the use of MM services remains the first and most common choice because of their excellent medical systems and rapid results [51,52]. It is of great necessity to improve the knowledge of unique advantages of TCM, such as safety, lower cost, effectiveness, convenience, and to strengthen the application of TCM in NCDs treatment [33].

In terms of travel time, all other things being equal, respondents were less likely to choose healthcare services with low accessibility in hypothesis of a minor disease scenario. For conditions perceived as minor, they would be willing to pay 102.84 CNY (15.43USD) for a reduction in travel time to below 30 min. Elderly people consist of the majority of patients with NCDs in China [53]. It has been observed that a less preferred option became more attractive when the convenience of healthcare service was improved for elderly patients in practice [18]. This may be linked to elderly patients’ poor physical condition. As a result, elderly patients with better accessibility to PHC facilities tended to choose PHC facilities as the first-contact care in hypothetical mild disease [54]. It is imperative to ensure that patients have accessible, affordable, and high-quality PHC. While travel time mattered less when health status was worsen-off [55,56]. The irrational condition of healthcare seeking would increase with the increasing uncertainty of disease expectation. They tend to choose top tertiary hospitals equipped with specialized medical resources and highly specialized medical experts [19]. This finding differed from a study regarding the health-seeking behavior of rural residents, which demonstrated that travel time was considered even more important when a resident’s health condition was perceived as severe [19]. One possible reason may be associated with the disparity between rural and urban areas in terms of healthcare accessibility. 

Consistent with previous studies, our study confirmed that cost had a significant impact on healthcare choices [57,58]. It should also be noted that cost was not rated as more important than other attributes. A study conducted at a tertiary hospital in Fuzhou showed that more than 50% of patients would choose a tertiary hospital in healthcare seeking, even if they pay medical expenditures by themselves completely [59]. This implies that although economic leverage has a certain impact on patient choice of medical treatment, financial incentives alone may not be enough to guide them to PHC facilities for healthcare utilization. Further qualitative studies are called for to explore the intrinsic factors.

Apart from these, care provider and type of service were also important for respondents to choose PHC facilities. Under hypothetical mild disease scenario, these two attributes were insignificant. Respondents rated specialized service and senior medical practitioner as positive attributes under a severe condition. WTP related to the change from general service to specialized service and from junior medical practitioner to senior medical practitioner were highly valued by respondents, worth 107.3 CNY (16.10 USD) and 565.8 CNY (84.87 USD), respectively. Intuitively, this occurred due to relatively minor symptoms and they did not need first-class medical technology. In addition, given that patients with NCDs have long-term nature disease and healthcare utilization, control of disease is more suitable in PHC facilities. Current studies also stated that many patients remain dissatisfied with the care provided by PHC facilities [58]. Sometimes patients bypass these facilities because they do not trust healthcare provided by PHC facilities, or they are not satisfied with the quality of care. In addition, few PHC facilities providers in China have the qualification to serve as gatekeepers or provide high-quality services [54]. Although training programs have increased the number of GPs in China, the number remains far below the nation’s needs [60]. Specialists from higher level hospitals should be motivated to provide services and to organize GPs at the grassroots level periodically. Promoting system of family doctor, establishing supporting measures which strengthen the construction of PHC should be considerations for government.

Some limitations should be noted in this study. First, our study analyzes stated preferences of the respondents to hypothetical scenarios, which may not necessarily reflect the choices they would make in a real setting. Consistency between revealed preferences and stated preferences should be explored in future research. Second, since PHC facilities’ decision-making is complex and these five key attributes in the design may not fully reflect respondents’ decisions in the real world. Finally, potential bias may exist in our study due to nearly two-thirds of respondents being female. While the research was supported by the local health bureau, the contact databases which were available for us to invite participants only represent specific population groups registered with family doctors. The risk of NCDs in women is higher than men in China [61,62] and sampling bias cannot be ruled out. This may limit the generalizability of the results to a wider population.

## 5. Conclusions

Our study is the first attempt to examine preferences for PHC facilities by patients with NCDs in China. Our results highlighted the relative importance of PHC facilities and patients’ WTP for each attribute level. The findings could provide evidence to inform PHC delivery, improve the participation of patients in PHC, and provide some evidence to promote shared decision-making.

## Figures and Tables

**Table 1:** DCE Attributes and Attribute Levels.

Hypothetical Scenarios		Explanation
Disease severity	Minor	Suppose that you are getting a cold, cough and so on
	Severe	Suppose that health status seriously affects your daily life over a long period of time and makes you worry and anxious
**Attributes**	**Levels**	**Explanation**
1. Type of service	General service ^a^ Specialized service	Type of service refers to different modes of services consisting of differing skill sets.
2. Treatment measures	Traditional Chinese Medicine (TCM) ^a^ Modern Medicine (MM) Integration of Traditional Chinese Medicine & Modern Medicine (Integration of TCM & MM)	Treatment measurements are different approaches (usually stand for differing medicine system) of diagnosis and treatment of diseases.
3. Cost (CNY) ^b^	100 ^a^ 200 300	Cost is average expense of each visit for healthcare seeking, which was set according to China Statistical Yearbook in 2017.
4. Travel time (min)	≤30 ^a^ >30	Travel time stands for the time taken to go to healthcare facilities from home (one-way travel).
5. Care provider	Senior medical practitioner ^a^ Junior medical practitioner	Care provider is the healthcare provider with differing seniority.

Notes: ^a^ Reference level for each attribute. ^b^ 1 CNY = 0.15 USD in 2018 (According to the International Monetary Fund, https://www.imf.org/external/index.htm).

**Table 2:** Respondents’ characteristics (n = 196).

Characteristic	N (%)
Gender	
Female	132 (67.4) (pre-defined quota: 49%)
Male	64 (32.6) (pre-defined quota: 51%)
Region	
Urban area	115 (58.7) (pre-defined quota: 69.1%)
Suburban area	81 (41.3) (pre-defined quota: 30.9%)
Age	
45–65	78 (39.8)
≥65	118 (60.2)
Marital status	
Single	26 (13.3)
Married	170 (86.7)
Education	
Elementary school and below	31 (15.8)
Middle school	100 (51.0)
High school and above	65 (33.2)
Employment	
Employed/Working	36 (18.4)
Retiree/Pensioner	118 (60.2)
Not working	42 (21.4)
Family per capita monthly income (CNY)	
≤1500	18 (9.2)
1500–4000	117 (59.7)
>4000	61 (31.1)

**Table 3:** Mixed logit main-effect model estimates and willingness to pay in hypothetical minor disease and severe disease scenarios.

		Minor Disease Scenario					Severe Disease Scenario				
Attributes and Levels		Mean	SE	SD	SE	WTP/95%CI	Mean	SE	SD	SE	WTP/95%CI
Type of service:											
General service ^a^		−0.03	0.05	0.03	0.28	−3.45 (−16.51, 9.61)	−0.20	0.08 *	0.64	0.11 **	−53.65 (−107.53, 0.24)
Specialized service		0.03	0.05	0.03	0.28	3.45 (−9.61, 16.51)	0.20	0.08 *	0.64	0.11 **	53.65 (−0.24, 107.53)
Treatment measures:											
TCM ^a^		−0.69	0.15 **	1.62	0.17 **	−94.15 (−135.91, −53.45)	−1.51	0.25 **	2.19	0.27 **	−400.71 (−610.50, −190.91)
MM		0.64	0.14 **	1.60	0.16 **	87.60 (47.73, 127.47)	1.35	0.22 **	2.18	0.26 **	361.02 (168.12, 553.93)
Integration of TCM&MM		0.05	0.07	0.01	0.32	6.55 (−13.47, 26.57)	0.16	0.11	0.18	0.30	42.75 (−14.35, 99.84)
Travel time:											
≤30 min ^a^		0.38	0.07 **	0.62	0.09 **	51.42 (32.09, 69.34)	0.05	0.07	0.45	0.11 **	12.16 (−26.37, 50.68)
>30 min		−0.38	0.07 **	0.62	0.08 **	−51.42 (−70.21, −32.64)	−0.05	0.07	0.45	0.11 **	−12.16 (−50.68, 26.37)
Care provider:											
Junior medical practitioner ^a^		0.10	0.06	0.55	0.09 **	13.14 (−3.70, 28.41)	−1.06	0.14 **	1.15	0.15 **	−282.90 (−439.37, −141.69)
Senior medical practitioner		−0.10	0.06	0.52	0.08 **	−13.14 (−29.40, 3.12)	1.06	0.14 **	1.10	0.14 **	282.90 (144.77,421.04)
Cost (CNY)		−0.007	0.001 **				−0.004	0.001 **			
Model fit	AIC	1650.13					1395.14				
	BIC	1716.68					1461.68				
	Log likelihood	−814.07					−686.57				
	Respondents, n	196									
	Observations, n	3136									

Notes: * *p* < 0.05, ** *p* < 0.01. ^a^ reference level for each attribute. Mean—the average preferences of the study population. SE—standard errors. SD—standard deviation. WTP—willingness to pay. AIC: Akaike information criterion, BIC: Bayesian information criterion.

**Table 4:** Results of the preference heterogeneity analysis.

Attributes and Levels	Minor Disease Scenario		Severe Disease Scenario	
	Mean	95%CI	Mean	95%CI
Type of service:				
General service ^a^				
Specialized service	0.0002	(−0.71, 0.71)	−0.35	(−1.64, 0.93)
Treatment measures:				
TCM ^a^				
MM	1.28	(−0.82, 3.39)	2.49	(−0.79, 5.77)
Integration of TCM&MM	0.07	(−1.03, 1.17)	1.04	(−0.56, 2.64)
Travel time:				
≤30 min ^a^				
>30 min	−0.06	(−1.02, 0.90)	−0.65	(−1.92, 0.62)
Care provider:				
Junior medical practitioner ^a^				
Senior medical practitioner	0.58	(−0.31, 1.47)	0.72	(−0.83,2.27)
Cost (CNY)	0.01	(−0.003, 0.02)	0.01	(−0.004, 0.02)
Interaction: attribute * demographic attributes				
Cost * region	−0.005 **	(−0.008, −0.001)		
Cost * marital status			−0.01 **	(−0.020, −0.003)
Cost * family per capita monthly income			0.006 **	(0.002, 0.010)
>30 min * region	−0.64 **	(−0.94, −0.33)		
Senior medical practitioner * gender	−0.28 *	(−0.560, −0.002)		
Senior medical practitioner * region			0.57 *	(0.06, 1.08)
Senior medical practitioner * marital status			0.92 *	(0.15, 1.68)
Specialized service * education	0.32 *	(0.05, 0.60)		
Specialized service * region			0.47 *	(0.07, 0.86)
MM * gender			−1.07 *	(−2.09, −0.06)
Integration of TCM&MM * employment			0.87 *	(0.06, 1.67)
Model fit AIC	1653.43		1411.46	
BIC	1974.08		1732.08	
Log likelihood	−773.71		−652.73	
Respondents, n	196			
Observations, n	3136			

Notes: * *p* < 0.05, ** *p* < 0.01. ^a^ reference level for each attribute. Mean—the average preferences of the study population. AIC: Akaike information criterion, BIC: Bayesian information criterion. For conciseness, only the significant interaction terms at 5% level are listed in the table.

## Data Availability

The data might be reached by contacting the correspondence author.

## Supplementary Materials

The following are available online at https://www.mdpi.com/1660-4601/17/11/3987/s1.

## Abbreviations

PHC	Primary healthcare
DCE	Discrete choice experiment
WTP	Willingness-to-pay
NCDs	Non-communicable diseases
MM	Modern Medicine
TCM	Traditional Chinese Medicine
Integration of TCM & MM	Integration of Traditional Chinese Medicine & Modern Medicine
GP	General practitioner
DALYs	Disability-adjusted life years
AIC	Akaike information criterion
BIC	Bayesian information criterion
SE	Standard errors
SD	Standard deviation
CI	Confidence intervals

## Appendix A

**Table A1:** Gross domestic product by district in Wuhan (2017).

District	Gross Domestic Product (100 Million CNY)
Jiangan	1073.60
Jianghan	1142.58
Qiaokou	668.07
Hanyang	948.31
Wuchang	1102.50
Qingshan	521.88
Hongshan	957.73
Wuhan Airport Economic Development Zone	730.63
Caidian	397.65
Jiangxia	770.98
Huangpi	702.49
Xinzhou	676.32

Notes: The statistics are based on Wuhan Statistical yearbook—2018.

## Appendix B

**Table A2:** Variables for the sociodemographic characteristics.

Demographic Attribute	Variables
Age	Continuous
Region	Urban ^a^
	Suburban
Gender	Male ^a^
	Female
Marital status	Single ^a^
	Married
Education	Middle school and below ^a^
	High school or higher
Employment	Working ^a^
	Not working
Family per capita monthly income (CNY)	≤3000 ^a^
	>3000

^a^ Reference level.
