# Supplementary material for: Preferences of Patients with Non-Communicable Diseases for Primary Healthcare Facilities: A Discrete Choice Experiment in Wuhan, China
Source: Int J Environ Res Public Health. 2020 Jun 4;17(11):3987. doi: 10.3390/ijerph17113987 (PMC7311994; doi:10.3390/ijerph17113987)
Supplement: Supplementary file 1 [file ijerph-17-03987-s001.zip › Supplementary files/Questionnaire Block 2.docx]

**Survey on preferences for primary healthcare facilities**

(Block-2)

This survey is being carried out by researchers in School of Medicine and Health Management, Tongji Medical College, Huazhong University of Science and Technology. We would like to know which primary healthcare facility you personally would prefer to go when you perceived your health condition as minor or severe. To do this you will be asked to indicate which of the two alternative primary healthcare facilities you would choose in hypothetical scenarios. The answers you provide here will only be used for research purposes and you cannot be identified from your answers.

The facilities differ in terms of the following ways: type of service, treatment measures, cost (CNY), travel time (min), care provider. All other aspects of the two facilities are the same.

**Explanation of attributes and levels for primary healthcare facilities：**

| **Attributes** | **Levels** | **Explanation** |
| --- | --- | --- |
| Type of service | General service | Type of service refers to different modes of services consisting of differing skill sets |
|  | Specialized service |  |
| Treatment measures | Traditional Chinese Medicine (TCM) | Treatment measurements are different approaches (usually stand for differing medicine system) of diagnosis and treatment of diseases |
|  | Modern Medicine (MM) |  |
|  | Integration of Traditional Chinese Medicine & Modern Medicine (Integration of TCM & MM) |  |
| Cost (CNY) | 100 CNY | Cost is average expense of each visit for healthcare seeking, which was set according to China Statistical Yearbook in 2017 |
|  | 200 CNY |  |
|  | 300 CNY |  |
| Travel time (min) | ≤30 mins | Travel time stands for the time taken to go to nearest healthcare facilities from home (one-way travel) |
|  | >30 mins |  |
| Care provider | Senior medical practitioners | Care provider is the healthcare providers with differing seniority |
|  | Junior medical practitioners |  |

**Example of the choice set (*Please do not fill this one*)**

Suppose you are in the physical condition described in the following case. Under this premise, if you now have two options for primary healthcare facilities, Facility A and Facility B. These two options differ in terms of type of service, treatment measures, cost, travel time and care provider. Please choose the preferred choice according to your personal preferences and tick the corresponding boxes. When you make choices, please assume that the conditions are the same except for the five attributes listed.

| **Characteristics** | **Facility A** | **Facility B** |
| --- | --- | --- |
| Type of service | Specialized service | General service |
| Treatment measures | MM | MM |
| Cost (CNY) | 300 CNY | 100 CNY |
| Travel time | ≤30 mins | ≤30 mins |
| Care provider | Senior medical practitioner | Junior medical practitioner |
| Which facility would you choose? (Please tick only one box at the right) |  |  |

**Part 1：The questionnaire of discrete choice experiment（DCE）**

Suppose you have a cold, cough that occasionally makes you feel uncomfortable and does not seriously affect daily life, even without going to a doctor. Please make your preferred choice by putting a tick (√) in the appropriate box. **Please note that in each of the choice the characteristics of facilities change.**

**Project 1**

| **Characteristics** | **Facility A** | **Facility B** |
| --- | --- | --- |
| Type of service | General service | Specialized service |
| Treatment measures | MM | Integration of TCM & MM |
| Cost (CNY) | 300 CNY | 100 CNY |
| Travel time | ＞30 mins | ≤30 mins |
| Care provider | Senior medical practitioner | Junior medical practitioner |
| Which facility would you choose? (Please tick only one box at the right) |  |  |

**Project 2**

| **Characteristics** | **Facility A** | **Facility B** |
| --- | --- | --- |
| Type of service | General service | Specialized service |
| Treatment measures | TCM | MM |
| Cost (CNY) | 200 CNY | 300 CNY |
| Travel time | ≤30 mins | ＞30 mins |
| Care provider | Junior medical practitioner | Senior medical practitioner |
| Which facility would you choose? (Please tick only one box at the right) |  |  |

**Project 3**

| **Characteristics** | **Facility A** | **Facility B** |
| --- | --- | --- |
| Type of service | Specialized service | General service |
| Treatment measures | MM | Integration of TCM & MM |
| Cost (CNY) | 100 CNY | 200 CNY |
| Travel time | ≤30 mins | ＞30 mins |
| Care provider | Junior medical practitioner | Senior medical practitioner |
| Which facility would you choose? (Please tick only one box at the right) |  |  |

**Project 4**

| **Characteristics** | **Facility A** | **Facility B** |
| --- | --- | --- |
| Type of service | General service | Specialized service |
| Treatment measures | TCM | MM |
| Cost (CNY) | 100 CNY | 200 CNY |
| Travel time | ＞30 mins | ≤30 mins |
| Care provider | Junior medical practitioner | Senior medical practitioner |
| Which facility would you choose? (Please tick only one box at the right) |  |  |

**Project 5**

| **Characteristics** | **Facility A** | **Facility B** |
| --- | --- | --- |
| Type of service | General service | Specialized service |
| Treatment measures | Integration of TCM & MM | MM |
| Cost (CNY) | 300 CNY | 100 CNY |
| Travel time | ≤30 mins | ＞30 mins |
| Care provider | Junior medical practitioner | Senior medical practitioner |
| Which facility would you choose? (Please tick only one box at the right) |  |  |

**Project 6**

| **Characteristics** | **Facility A** | **Facility B** |
| --- | --- | --- |
| Type of service | General service | Specialized service |
| Treatment measures | MM | Integration of TCM & MM |
| Cost (CNY) | 100 CNY | 200 CNY |
| Travel time | ＞30 mins | ≤30 mins |
| Care provider | Junior medical practitioner | Senior medical practitioner |
| Which facility would you choose? (Please tick only one box at the right) |  |  |

**Project 7**

| **Characteristics** | **Facility A** | **Facility B** |
| --- | --- | --- |
| Type of service | General service | Specialized service |
| Treatment measures | TCM | MM |
| Cost (CNY) | 200 CNY | 300 CNY |
| Travel time | ＞30 mins | ≤30 mins |
| Care provider | Senior medical practitioner | Junior medical practitioner |
| Which facility would you choose? (Please tick only one box at the right) |  |  |

**Project 8**

| **Characteristics** | **Facility A** | **Facility B** |
| --- | --- | --- |
| Type of service | Specialized service | General service |
| Treatment measures | Integration of TCM & MM | TCM |
| Cost (CNY) | 100 CNY | 200 CNY |
| Travel time | ＞30 mins | ≤30 mins |
| Care provider | Senior medical practitioner | Junior medical practitioner |
| Which facility would you choose? (Please tick only one box at the right) |  |  |

Suppose health status seriously affects your daily life over a long period of time and makes you worry and anxious. Please make your preferred choice by putting a tick (√) in the appropriate box. **Please note that in each of the choice the characteristics of facilities change.**

**Project 1**

| **Characteristics** | **Facility A** | **Facility B** |
| --- | --- | --- |
| Type of service | General service | Specialized service |
| Treatment measures | MM | Integration of TCM & MM |
| Cost (CNY) | 300 CNY | 100 CNY |
| Travel time | ＞30 mins | ≤30 mins |
| Care provider | Senior medical practitioner | Junior medical practitioner |
| Which facility would you choose? (Please tick only one box at the right) |  |  |

**Project 2**

| **Characteristics** | **Facility A** | **Facility B** |
| --- | --- | --- |
| Type of service | General service | Specialized service |
| Treatment measures | TCM | MM |
| Cost (CNY) | 200 CNY | 300 CNY |
| Travel time | ≤30 mins | ＞30 mins |
| Care provider | Junior medical practitioner | Senior medical practitioner |
| Which facility would you choose? (Please tick only one box at the right) |  |  |

**Project 3**

| **Characteristics** | **Facility A** | **Facility B** |
| --- | --- | --- |
| Type of service | Specialized service | General service |
| Treatment measures | MM | Integration of TCM & MM |
| Cost (CNY) | 100 CNY | 200 CNY |
| Travel time | ≤30 mins | ＞30 mins |
| Care provider | Junior medical practitioner | Senior medical practitioner |
| Which facility would you choose? (Please tick only one box at the right) |  |  |

**Project 4**

| **Characteristics** | **Facility A** | **Facility B** |
| --- | --- | --- |
| Type of service | General service | Specialized service |
| Treatment measures | TCM | MM |
| Cost (CNY) | 100 CNY | 200 CNY |
| Travel time | ＞30 mins | ≤30 mins |
| Care provider | Junior medical practitioner | Senior medical practitioner |
| Which facility would you choose? (Please tick only one box at the right) |  |  |

**Project 5**

| **Characteristics** | **Facility A** | **Facility B** |
| --- | --- | --- |
| Type of service | General service | Specialized service |
| Treatment measures | Integration of TCM & MM | MM |
| Cost (CNY) | 300 CNY | 100 CNY |
| Travel time | ≤30 mins | ＞30 mins |
| Care provider | Junior medical practitioner | Senior medical practitioner |
| Which facility would you choose? (Please tick only one box at the right) |  |  |

**Project 6**

| **Characteristics** | **Facility A** | **Facility B** |
| --- | --- | --- |
| Type of service | General service | Specialized service |
| Treatment measures | MM | Integration of TCM & MM |
| Cost (CNY) | 100 CNY | 200 CNY |
| Travel time | ＞30 mins | ≤30 mins |
| Care provider | Junior medical practitioner | Senior medical practitioner |
| Which facility would you choose? (Please tick only one box at the right) |  |  |

**Project 7**

| **Characteristics** | **Facility A** | **Facility B** |
| --- | --- | --- |
| Type of service | General service | Specialized service |
| Treatment measures | TCM | MM |
| Cost (CNY) | 200 CNY | 300 CNY |
| Travel time | ＞30 mins | ≤30 mins |
| Care provider | Senior medical practitioner | Junior medical practitioner |
| Which facility would you choose? (Please tick only one box at the right) |  |  |

**Project 8**

| **Characteristics** | **Facility A** | **Facility B** |
| --- | --- | --- |
| Type of service | Specialized service | General service |
| Treatment measures | Integration of TCM & MM | TCM |
| Cost (CNY) | 100 CNY | 200 CNY |
| Travel time | ＞30 mins | ≤30 mins |
| Care provider | Senior medical practitioner | Junior medical practitioner |
| Which facility would you choose? (Please tick only one box at the right) |  |  |

**Part 2: Respondents’ socio-demographic characteristics**

**1. Gender:**  Male Female

**2. Age: ____**

**3. Region:** Urban area Suburban area

**4. Marital status:** Unmarried Married Divorced/Separated/Widowed

**5.** **Education:**

Elementary school and below Middle school High school and above

**6. Employment：**

Employed/working Not working Retiree/pensioner

**7. Family per capita monthly income (CNY): ____**

**Thank you for taking part in this survey.**
